# R292K Substitution and Drug Susceptibility of Influenza A(H7N9) Viruses

**DOI:** 10.3201/eid1909.130724

**Published:** 2013-09

**Authors:** Katrina Sleeman, Zhu Guo, John Barnes, Michael Shaw, James Stevens, Larisa V. Gubareva

**Affiliations:** Centers for Disease Control and Prevention, Atlanta, Georgia, USA

**Keywords:** H7N9, avian influenza, neuraminidase inhibitor, zanamivir, oseltamivir, R292K, recombinant neuraminidase, influenza, antimicrobial resistance, antiviral resistance, viruses

## Abstract

Neuraminidase inhibitors are the only licensed antiviral medications available to treat avian influenza A(H7N9) virus infections in humans. According to a neuraminidase inhibition assay, an R292K substitution reduced antiviral efficacy of inhibitors, especially oseltamivir, and decreased viral fitness in cell culture. Monitoring emergence of R292K-carrying viruses using a pH-modified neuraminidase inhibition assay should be considered.

The recent emergence of an avian influenza A(H7N9) virus causing human infections in China (*1*,*2*) is of global concern. Most patients infected during this outbreak have experienced severe disease and required hospitalization; the mortality rate is 21% (*3*). Although epidemiologic investigations have revealed no evidence of sustained human-to-human transmission (*4*), suspected limited human-to-human transmission has been reported (*3*).

As with any emergent influenza virus, it is critical to assess the susceptibility of the influenza A(H7N9) outbreak virus to antiviral drugs, which are the first line of defense before an effective vaccine becomes available. Two classes of antiviral drugs are approved for management of influenza A infections, neuraminidase (NA) inhibitors (NAIs) and matrix 2 protein (M2) blockers (adamantanes). The outbreak viruses carry the established adamantane resistance marker, an S31N substitution in the M2 protein (*2*), leaving NAIs as the only licensed treatment option. Among the 4 NAIs, oseltamivir and zanamivir are approved in many countries; peramivir has been approved in Japan, South Korea, and China; and laninamivir is approved only in Japan. In contrast to those for adamantanes, genetic markers of resistance to NAIs are often subtype specific and drug specific (*5*). Therefore, monitoring drug susceptibility of the influenza A(H7N9) viruses requires testing in phenotypic assays using all available NAIs.

## The Study

Our aim was to assess NAI susceptibility of 2 influenza A(H7N9) outbreak virus isolates provided by the Chinese Center for Disease Control and Prevention. The influenza A/Anhui/1/2013 isolate was recovered from an untreated patient and contained no notable NAI-resistance markers in the NA gene. When tested in the NA inhibition (NI) assay (*6*), the virus yielded subnanomolar IC_50_s (concentration of neuraminidase inhibitor required to reduce enzyme activity by 50%) with all 4 NAIs, similar to results for the drug-sensitive seasonal influenza A viruses used as controls (Table 1). The second isolate, influenza A/Shanghai/1/2013, was collected from a patient who had received 2 doses of oseltamivir; the isolate was reported to contain an NA substitution, R292K (*2*). R292K is known to alter NAI susceptibility in viruses of N2 (*7*) and N9 (*8*) subtypes. However, A/Shanghai/1/2013 virus was reported to be susceptible to both oseltamivir and zanamivir on the basis of NI assay data (*2*). To clarify the effect of R292K on NAI susceptibility of influenza A(H7N9) viruses, the A/Shanghai/1/2013 egg-grown isolate (E1) was received and tested at the US Centers for Disease Control and Prevention by using the NI assay (*6*). Our data showed full susceptibility of A/Shanghai/1/2013 virus to oseltamivir (Table 1), an observation consistent with a previous report (*2*). Analysis of the E1 isolate by pyrosequencing assay (*9*) revealed a polymorphism at NA residue 292, containing arginine (23%) and lysine (77%; Table 1). Further analysis of the E1 isolate by PacBio deep sequencing confirmed that 77% of the virus population possessed the lysine 292 variant (Table 1).

**Table 1:** Susceptibility of influenza viruses to neuraminidase inhibitors, according to NI assay*

Sample type	Subtype	Virus name (passage)	AA at 292†	% K292	IC_50_ nmol/L (-fold)			
					Oseltamivir	Zanamivir	Peramivir	Laninamivir
Virus isolate	H7N9	A/Anhui/1/2013 (E2/S1)	R		0.17 (1)	0.33 (1)	0.06 (1)	0.46 (1)
		A/Shanghai/1/2013 (E1)	R and K	77	0.59 (3)	NT	NT	NT
Recombinant NA	H7N9	A/Anhui1/2013	R		0.25 (1)	0.52 (2)	0.07 (1)	0.60 (1)
			K	100	5153 (>1,000)	28.05 (54)	127.60 (>1,000)	17.53 (29)
		A/Shanghai/1/2013	R		0.25 (1)	0.46 (1)	0.06 (1)	0.44 (1)
			K	100	4987 (>1,000)	23.46 (51)	101.89 (>1,000)	15.35 (35)
Reference virus	H3N2	Oseltamivir-sensitive A/Washington/01/2007	–		0.07 (1)	0.23 (1)	0.08 (1)	0.29 (1)
		Oseltamivir-resistant A/Bethesda/956/2006	K	100	3974 (>1,000)	6.83 (30)	16.27 (203)	2.51 (9)
	H1N1‡	Oseltamivir-sensitive A/California/12/2012	–		0.19 (1)	0.16 (1)	0.06 (1)	0.17 (1)
		Oseltamivir-resistant A/Texas/23/2012	–		157.25 (828)	0.20 (1)	15.81 (264)	0.26 (2)

*NI, neuraminidase inhibition; AA, amino acid; IC_50_, concentration of neuraminidase inhibitor required to reduce enzyme activity by 50%; E, passage in eggs; S, passage in MDCK-SIAT1 cells (/ separates passage before and after arrival to CDC); NA, neuraminidase; NT, not tested; RT-PCR, reverse transcription PCR. †AA position: R292K (N2 numbering); R294K (straight full-length N9 numbering). Single-nucleotide polymorphism analysis was performed by using the pyrosequencing assay (RT-PCR primers: N9-F731-Bio, 5’-CT GGA CCT GCA GAC ACA AGA ATA-3’, N9-R926, 5’-TGT GTC ATT GCT ACT GGG TCT ATC-3’; sequencing primer: N9–292/294-R889-seq, 5’-TAT TTG AGC CCT GCC-3’) and confirmed by deep sequencing. Pac bio RS sequencing library was constructed by using a 701-bp RT-PCR amplicon generated by RT-PCR (N9NA-F204,5’- CAACATCCAAATGGAAGAGAGAA-3’; N9NA-R903 5’-TGTGTCATTGCTACTGGGTCTATC-3’). A single v3 SMRT cell was used for each library, and data were collected on 2 × 55 min movies. Only circular consensus sequencing reads were used in the analysis. Subpopulation detection was analyzed by using CLC Genomics Workbench version 6.01 (CLC Bio, Aarhus, Denmark). Isolates were tested in the NI assay by using the NA-Fluor kit (6). Fold change in IC_50_- compared with drug-sensitive subtype-specific control. IC_50_ values represent the average taken from at least 4 replicates, with the exception of A/Shanghai/1/2013 (E1), because of insufficient sample volume. Oseltamivir-susceptible and oseltamivir-resistant reference viruses were used as controls in NI assays. Oseltamivir refers to oseltamivir carboxylate. Reference virus A/Texas/23/2012 contains H275Y oseltamivir-resistance conferring neuraminidase substitution. ‡Pandemic influenza A(H1N1) 2009 virus.

The inability to detect changes in oseltamivir IC_50_ despite the presence of R292K raised 2 questions: are conventional NI assays sufficiently sensitive to detect oseltamivir resistance caused by R292K, and is R292K truly a marker of oseltamivir resistance when it is present in these A(H7N9) outbreak viruses? We hypothesized that failure to detect the oseltamivir-resistant population by using the NI assay may stem from substantially reduced activity of the R292K variant NA. Previous studies have shown that the optimal pH for R292K enzyme activity is ≈5.3 (*7*), whereas the conventional NI assay uses a buffer at pH 6.5. We retested A/Shanghai/1/2013 (E1) by using the NI assay under the lower pH condition. The E1 isolate exhibited a higher oseltamivir IC_50_ (643 nmol/L vs. 0.6 nmol/L; Table 2) than that determined by the conventional assay, a finding consistent with our hypothesis. IC_50_s of A/Anhui/1/2013 and reference viruses were either unchanged or found to increase slightly at the lower pH (Table 2).

**Table 2:** Susceptibility to neuraminidase inhibitors according to modified NI assay with pH 5.1*

Sample type	Subtype	Virus name (passage)	AA at 292†	% K292	IC_50_ nmol/L (fold)			
					Oseltamivir	Zanamivir	Peramivir	Laninamivir
Virus Isolate	H7N9	A/Anhui/1/2013 (E2/S1)	R		0.90 (1)	1.00 (1)	0.12 (1)	1.29 (1)
		A/Shanghai/1/2013 (E1)	R and K	77	642.78 (714)	NT	NT	NT
Recombinant NA	H7N9	A/Anhui1/2013	R		0.53 (1)	0.85 (1)	0.09 (1)	0.92 (1)
			K	100	9,078 (>1,000)	54.18 (56)	89.70 (814)	30.50 (25)
		A/Shanghai/1/2013	R		1.08 (1)	1.45 (1)	0.19 (1)	1.90 (1)
			K	100	8,351 (>1,000)	53.36 (53)	77.01 (642)	29.72 (23)
Reference virus	H3N2	Oseltamivir-sensitive A/Washington/01/2007	–		0.68 (1)	0.45 (1)	0.10 (1)	0.86 (1)
		Oseltamivir-resistant A/Bethesda/956/2006	K	100	6,083 (>1,000)	14.57 (32)	8.58 (86)	5.65 (7)
	H1N1‡	Oseltamivir-sensitive A/California/12/2012	–		0.74 (1)	0.23 (1)	0.07 (1)	0.21 (1)
		Oseltamivir-resistant A/Texas/23/2012	–		364.74 (493)	0.31 (1)	30.17 (431)	0.42 (2)

*NI, neuraminidase inhibition; AA, amino acid; IC_50_, concentration of neuraminidase inhibitor required to reduce enzyme activity by 50%; E, passage in eggs; S, passage in MDCK-SIAT1 cells (/ separates passage before and after arrival to CDC); NA, neuraminidase; NT, not tested; RT-PCR, reverse transcription PCR. †AA position: R292K (N2 numbering); R294K (straight full-length N9 numbering). Single-nucleotide polymorphism analysis was performed by using the pyrosequencing assay (RT-PCR primers: N9-F731-Bio, 5’-CT GGA CCT GCA GAC ACA AGA ATA-3’, N9-R926, 5’-TGT GTC ATT GCT ACT GGG TCT ATC-3’; sequencing primer: N9–292/294-R889-seq, 5’-TAT TTG AGC CCT GCC-3’) and confirmed by deep sequencing. Pac bio RS sequencing library was constructed by using a 701bp RT-PCR amplicon generated by RT-PCR (N9NA-F204, 5’-CAACATCCAAATGGAAGAGAGAA-3’; N9NA-R903 5’-TGTGTCATTGCTACTGGGTCTATC-3’). A single v3 SMRT cell was used for each library and data was collected on 2 × 55 min movies. Only circular consensus sequencing reads were used in the analysis. Subpopulation detection was analyzed by using CLC Genomics Workbench version 6.01 (CLC Bio, Aarhus, Denmark). Isolates were tested in the NI assay by using the NA-Fluor kit (6). Fold change in IC_50_ compared with drug-sensitive subtype-specific control. IC_50_ values represent the average taken from at least 4 replicates, with the exception of A/Shanghai/1/2013 (E1), due to insufficient sample volume. Oseltamivir-susceptible and oseltamivir-resistant reference viruses were used as controls in NI assays. Oseltamivir refers to oseltamivir carboxylate. Reference virus A/Texas/23/2012 contains H275Y oseltamivir,resistance conferring neuraminidase substitution. ‡Pandemic influenza A(H1N1) 2009 virus.

As part of further investigation of the role of R292K in altering NAI susceptibility, recombinant NA proteins (rNAs) of A/Shanghai/1/2013 isolate and A/Anhui/1/2013 isolate were expressed in insect cells by using a transient expression system. The rNAs were tested with 4 NAIs in conventional and pH-modified NI assays (Tables 1, 2). Irrespective of the assay and N9 backbone used, oseltamivir showed an inhibitory effect on the R292K rNAs activity only at concentrations >1,000 nmol/L. The R292K rNAs also showed increased IC_50_s for peramivir, zanamivir, and laninamivir (Tables 1, 2), consistent with previous findings (*5*). IC_50_s of the NAIs for the rNAs lacking R292K were comparable with those for the A/Anhui/1/2013 virus.

The NA activity of the rNAs was tested at multiple pH points in MES buffer supplemented with 4 mmol/L CaCl_2_. Activity of the R292K rNA peaked at pH 5.1 and increased by 5-fold compared with that measured under conventional assay conditions (pH 6.5). Conversely, the NA activity of rNA lacking this change was almost unchanged across the pH range tested (pH 4.9−6.9). These findings indicate that the R292K virus population could be concealed because of its reduced enzymatic activity under conventional assay conditions. NI assays with rNA proteins can clarify the extent of NAI sensitivity for each virus mutant and should be considered when analyzing heterogeneous virus populations with suspected NAI resistance.

To interpret NI assay results, criteria from the World Health Organization Antiviral Working Group were applied (*10*), and comparative differences in IC_50_s (which defines inhibition as normal [<10], reduced [10–100] or highly reduced [>100]) were determined by using a subtype-specific reference. The A/Shanghai/1/2013 (E1) isolate exhibited highly reduced inhibition by oseltamivir at pH 5.1. On the basis of data obtained by using rNAs, the R292K conferred highly reduced inhibition by peramivir, in addition to oseltamivir, and reduced inhibition by zanamivir and laninamivir (Tables 1,2).

## Conclusions

R292 is a highly conserved amino acid across all NA subtypes, and together with 2 other highly conserved residues (R118 and R371), it forms an arginine triad in the enzyme active site (*5*). R292K is a rare substitution and to date has only been reported in viruses collected from patients treated with oseltamivir (*2*,*5*). In addition to A/Shanghai/1/2013 isolate, there is evidence of additional influenza A(H7N9) isolates with the R292K substitution (*11*). In this study, propagation of A/Shanghai/1/2013 (E1) isolate in eggs and in MDCK-SIAT1 cells resulted in reversion to wild-type (23% Arg in E1 to 100% in E1/S3), confirming results of previous studies with N2 subtype viruses (*12*). Therefore, fitness of the A/Shanghai/1/2013 R292K virus is probably compromised when replication occurs in the absence of an NAI. However, propagation of the E1 isolate in the presence of oseltamivir (100 nmol/L) resulted in enrichment of the R292K population (from 77% to 100%), demonstrating a growth advantage over the wild-type.

Replication of the E1 isolate in the presence of any NAI in cell culture might lead to enrichment with R292K, because even a small growth advantage would reduce the proportion of the wild type. The efficacy of NAIs in clinical management of influenza (H7N9) infection remains unknown and may be compromised to a certain extent when R292K is present. Animal model studies are needed to aid in the understanding of clinical relevance of R292K. Reduction of NA activity caused by R292K may detrimentally affect transmission of the virus, as indicated by an R292K influenza A(H3N2) virus that showed reduced infectivity in mice (*13*,*14*) and ferrets (*12*,*13*,*15*) and was not transmitted among ferrets (*12*,*15*). The data reported here demonstrate the continued importance of monitoring drug susceptibility in emergent influenza viruses and highlight the challenges involved in laboratory assessment of NAI drug susceptibility testing.
